# MicroRNA-22 (miR-22) Overexpression Is Neuroprotective via General Anti-Apoptotic Effects and May also Target Specific Huntington’s Disease-Related Mechanisms

**DOI:** 10.1371/journal.pone.0054222

**Published:** 2013-01-17

**Authors:** Ana Jovicic, Julien Francisco Zaldivar Jolissaint, Roger Moser, Mariana de Fatima Silva Santos, Ruth Luthi-Carter

**Affiliations:** 1 Brain Mind Institute, École Polytechnique Fédérale de Lausanne, Lausanne, Vaud, Switzerland; 2 Department of Cell Physiology and Pharmacology, University of Leicester, Leicester, United Kingdom; The Chinese University of Hong Kong, Hong Kong

## Abstract

**Background:**

Whereas many causes and mechanisms of neurodegenerative diseases have been identified, very few therapeutic strategies have emerged in parallel. One possible explanation is that successful treatment strategy may require simultaneous targeting of more than one molecule of pathway. A new therapeutic approach to have emerged recently is the engagement of microRNAs (miRNAs), which affords the opportunity to target multiple cellular pathways simultaneously using a single sequence.

**Methodology/Principal Findings:**

We identified miR-22 as a potentially neuroprotective miRNA based on its predicted regulation of several targets implicated in Huntington’s disease (histone deacetylase 4 (HDAC4), REST corepresor 1 (Rcor1) and regulator of G-protein signaling 2 (Rgs2)) and its diminished expression in Huntington’s and Alzheimer’s disease brains. We then tested the hypothesis that increasing cellular levels of miRNA-22 would achieve neuroprotection in *in vitro* models of neurodegeneration. As predicted, overexpression of miR-22 inhibited neurodegeneration in primary striatal and cortical cultures exposed to a mutated human huntingtin fragment (Htt171-82Q). Overexpression of miR-22 also decreased neurodegeneration in primary neuronal cultures exposed to 3-nitropropionic acid (3-NP), a mitochondrial complex II/III inhibitor. In addition, miR-22 improved neuronal viability in an *in vitro* model of brain aging. The mechanisms underlying the effects of miR-22 included a reduction in caspase activation, consistent with miR-22′s targeting the pro-apoptotic activities of mitogen-activated protein kinase 14/p38 (MAPK14/p38) and tumor protein p53-inducible nuclear protein 1 (Tp53inp1). Moreover, HD-specific effects comprised not only targeting HDAC4, Rcor1 and Rgs2 mRNAs, but also decreasing focal accumulation of mutant Htt-positive foci, which occurred via an unknown mechanism.

**Conclusions:**

These data show that miR-22 has multipartite anti-neurodegenerative activities including the inhibition of apoptosis and the targeting of mRNAs implicated in the etiology of HD. These results motivate additional studies to evaluate the feasibility and therapeutic efficacy of manipulating miR-22 *in vivo*.

## Introduction

HD is a fatal hereditary neurodegenerative disease caused by a CAG repeat expansion mutation in exon 1 of the *Huntingtin* (*Htt*) gene, which results in a toxic polyglutamine (polyQ) stretch in the encoded Huntingtin (Htt) protein. The clinical features of disease include abnormal involuntary choreiform movements as well as mood and personality changes. Despite the monogenic nature of the HD, the disease demonstrates a complex etiology, likely due to the fact that mutant Htt resides in multiple cellular compartments and complexes, where it may disrupt organellar function and form protein aggregates [1]. Although the roles of various soluble and aggregated Htt species in the disease process remain to be elucidated, HD is widely considered to be a misfolded protein accumulation disorder and intracellular redistribution of Htt generally foretells its neurotoxicity. Specific cellular mechanisms implicated in HD pathogenesis include the dysregulation of transcription, cell signaling, and metabolism.

microRNAs (miRNAs) are 19–24 nucleotide noncoding RNAs that act as negative regulators of post-transcriptional gene expression [2], [3]. A single miRNA can target many mRNAs in parallel, comprising a potential way to simultaneously modify the activities of multiple pathways. If suitable endogenous or exogenous sequences could be found, miRNAs could comprise a rational new approach to the treatment of mechanistically complex diseases, including neurodegenerative diseases. In addition to their *a priori* potential for multipartite neuroprotective activities, the dysregulation of miRNA expression has been specifically implicated in the early pathogenesis of neurodegenerative diseases [4], including Huntington’s disease (HD) [5]–[8]. Therefore, restoring endogenous levels of miRNA expression might also be used to slow disease onset or progression. This has, for example, been shown in amyotrophic lateral sclerosis (ALS), where upregulation of miR-206 has been shown to be part of a cellular autocompensatory mechanism by promoting regeneration at neuromuscular synapses and slowing ALS progression through the downregulation of HDAC4 expression [9].

With the goal of identifying endogenous miRNA sequences that might simultaneously engage a range of HD therapeutic targets, we set out to identify brain miRNAs with a suitable target profile. We also took into account evidence for miRNA dysregulation in HD, and found one miRNA, miR-22, that fulfilled both criteria.

## Results

### miR-22 Targets Multiple Genes Involved in Huntington’s Disease

miR-22 is an miRNA that has previously been shown to be downregulated in both HD and Alzheimer’s disease brain [7], [10]. Using the miRNA target algorithm TargetScan, we discovered that miR-22 is predicted to target multiple mRNAs that have been implicated in HD pathogenesis. First, miR-22 is predicted to target Rcor1, a crucial regulator of neuronal gene expression; the Restrictive Element 1 Silencing Transcription Factor pathway has been shown to be hyperactive in HD, leading to a large-scale repression of neuronal genes in affected neurons [11]. Second, miR-22 is predicted to target Rgs2; we have recently shown that decreased Rgs2 expression in striatal neurons is protective in *in vitro* models of HD by decreasing extracellular-signal-regulated kinase (ERK) activation [12]. miR-22 was also predicted to target HDAC4; reversal of acetylation defects have been shown to ameliorate neurodegeneration in cellular and animal models of HD [13], and recent evidence has indicated that the administration of HDAC inhibitor suberoylanilide hydroxamic acid (SAHA) may work via diminishing HDAC4 expression post-transcriptionally [14].

**Figure 1:**
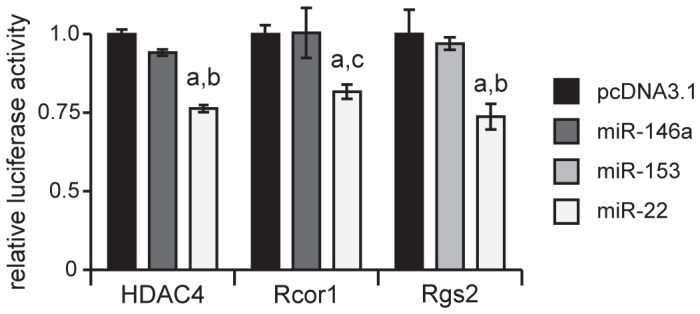
miR-22 targets Rcor1, Rgs2, and HDAC4 3′ UTRs. Luciferase constructs expressing the *Renilla* luciferase gene under the control of the corresponding 3′ UTRs were co-transfected in HEK293T cells with either a plasmid expressing miR-22 or an empty plasmid (pcDNA3.1). The intensity of *Renilla* luciferase luminescence was normalized to firefly luciferase luminescence intensity, which was not under the control of a 3′ UTR. Graphs represent mean values ± SD, n = 6. a indicates p-value <0.01, by unpaired t test compared to a control sample transfected with the empty vector pcDNA3.1. b indicates p<0.01, by unpaired t test compared to the control sample transfected with a miRNA not predicted to bind to the 3′ UTR (miR-146a or miR-153). c indicates p<0.05, by unpaired t test compared to the control sample transfected with a miRNA which is not predicted to bind to the 3′ UTR (miR-146a).

We therefore hypothesized that miR-22 might be a potential therapeutic approach to treating HD via tandem regulation of the above pathways. In order to first confirm the pertinent target predictions, we used 3′ untranslated region (UTR) luciferase assays to show that miR-22 specifically interacts with the 3′ UTRs of the Rcor1, Rgs2 and HDAC4 mRNAs (**Figure 1**), compared to control miRNAs that are not predicted to target these mRNAs (miR-146a served as the control for Rcor1 and HDAC4 whereas miR-153 served as a control for Rgs2 because Rgs2 contains a predicted miR-146a binding site).

We next tested the potential for miR-22 to achieve neuroprotection. In these experiments we assessed miR-22 overexpression in the test case to no miRNA expression as a control; we reasoned that this condition provided a high-stringency comparator, since either miRNA overexpression or exposure to additional lentiviral vector exposure could itself potentially compromise neuronal survival and confound the interpretation of our results. In other words, comparing the effect of miR-22 to the effect of another miRNA is problematic because it would be impossible to distinguish whether miR-22 were neuroprotective, or merely less toxic, than the other lentivirally-delivered miRNA.

**Figure 2:**
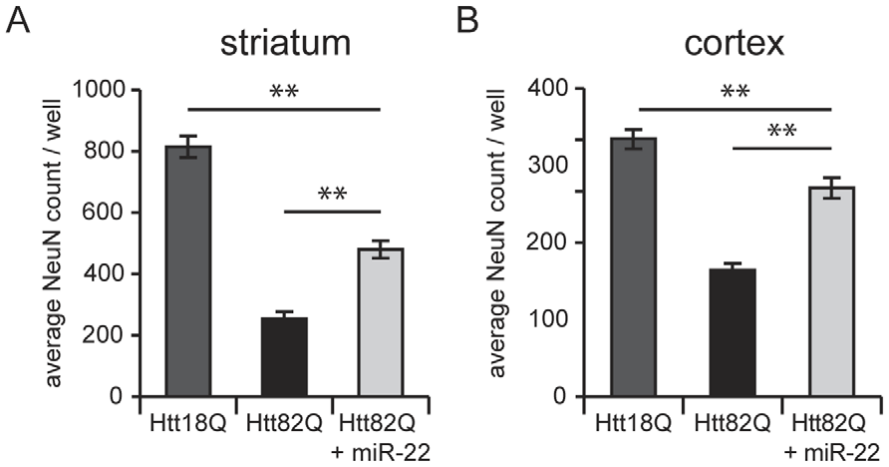
miR-22 is neuroprotective against mutant Htt. Primary striatal neurons A) or cortical neurons B) were infected on DIV1 with lentiviral vectors encoding wild-type (Htt18Q) or mutant (Htt82Q) Htt171 fragments under the control of the TRE promoter. On DIV4, where denoted, cultures were infected with vector encoding miR-22. Neuronal viability was assessed after 3 weeks in culture by quantification of NeuN-positive cells. Graphs represent mean ± SEM, n = 15. ** represents p-value <0.01.

We first assessed the effect of miR-22 overexpression in *in vitro* models of HD comprising lentiviral-mediated expression of mutant Htt fragments (Htt171-82Q versus Htt171-18Q) in primary rat striatal or cortical neurons [15], [16]; these models have previously been shown to exhibit HD-like neuropathologies and gene expression changes [17]. As expected, Htt171-82Q decreased the viability of both striatal and cortical neurons; moreover, as predicted viability of the neuronal cells was restored by miR-22 overexpression (**Figure 2**).

**Figure 3:**
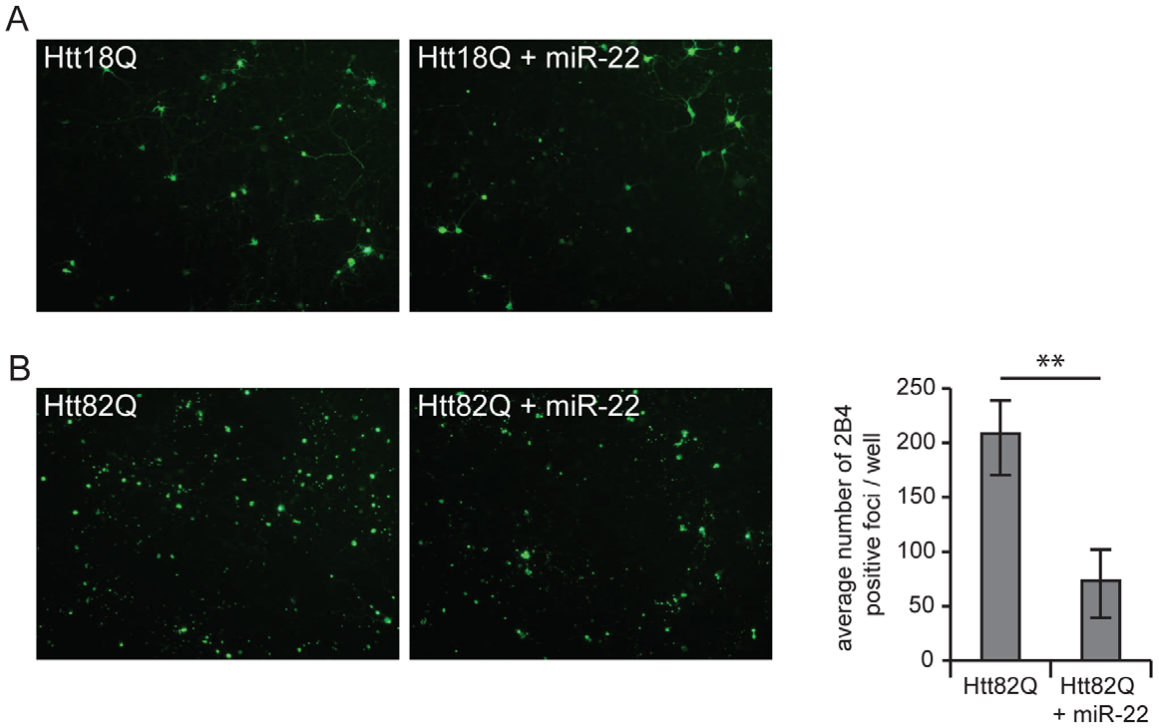
miR-22 decreases the focal accumulation of Htt protein. Primary striatal neurons were infected on DIV1 with lentiviral vectors encoding tTA and mutant (Htt82Q) Htt171 fragments under the control of the TRE promoter. Where indicated cells were co-infected on DIV4 with vector encoding miR-22. Htt accumulation was assessed using anti-Htt antibody 2B4. A) Numerous Htt-positive foci were detected in cells expressing Htt171-82Q. B) Fewer Htt-positive foci were detected in cells expressing miR-22+ Htt171-82Q. C) Htt-enriched foci were quantified using ImageJ software as described in Methods. Graphs represent mean ± SEM, n = 10. ** represents p-value <0.01, by unpaired t-test. Scale bar = 10 µm.

Although miR-22 is not predicted to target Htt, we nonetheless considered whether our observed disease-modifying effects might involve changes in Htt accumulation. This was assessed in parallel cultures immunostained with anti-Htt antibodies (see Methods), in which quantification of Htt-enriched foci of ≥2 µm (which might represent either compartmentalized of Htt accumulation or Htt-positive inclusions) was undertaken. To our surprise, the number of Htt-enriched foci was diminished by miR-22 (**Figure 3**). We were able to confirm this activity in multiple experiments, but have not yet been able to identify a predicted target to account for the mechanism of this effect. Nonetheless, the reduction of mutant Htt accumulation (or correction of abnormal Htt distribution) by miR-22 might contribute to its disease-mitigating activity in HD.

### miR-22 is Protective in Multiple *in vitro* Models of Neurodegeneration

**Figure 4:**
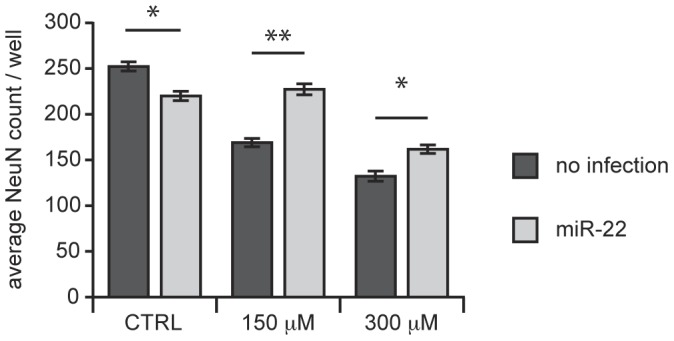
miR-22 is protective against 3-NP toxicity. Cultured primary striatal neurons (2.5 weeks *in vitro*) were treated with the indicated concentrations of 3-NP for 48 hours or left untreated (CTRL), and survival of neurons overexpressing miR-22 was compared to neurons not overexpressing miR-22 (uninfected). Cell viability was quantified after immunolabeling for the neuronal marker NeuN. Graphs represent mean ± SEM, n = 15. * represents p-value <0.05, ** represents p-value <0.01, by unpaired t-test.

In order to test whether non-Htt-targeting effects also contributed to the anti-neurodegenerative activity of miR-22, we employed a different HD model that would not involve Htt expression. Previous studies have shown that administration or accidental ingestion of 3-NP, an irreversible inhibitor of succinate dehydrogenase [18] (a component of the Krebs cycle and complex II/III of the mitochondrial electron transport system), produces neuropathologic and motor phenotypes similar to that of HD [19], [20]. We employed an acute *in vitro* version of this model, which involved treating striatal neurons with 3-NP for 48 hours [21]. We again chose to test the beneficial effects of miR-22 against the stringent control comprising neuronal cells without miRNA overexpression so to avoid confounding toxicities of the lentiviral vectors or control miRNA expression. Although the miR-22-expressing vector modestly decreased neuronal viability (NeuN-positive cell count) compared to the non-treated control (**Figure 4**, **left**, reflecting toxicity of the lentiviral vector application), miR-22 overexpression increased the survival of neurons treated with 3-NP (Figure 4, middle and right). The significant neuroprotection by miR-22, in this model indicated that the neuroprotective activities of miR-22 include mechanisms beyond regulating mutant Htt accumulation or distribution.

**Figure 5:**
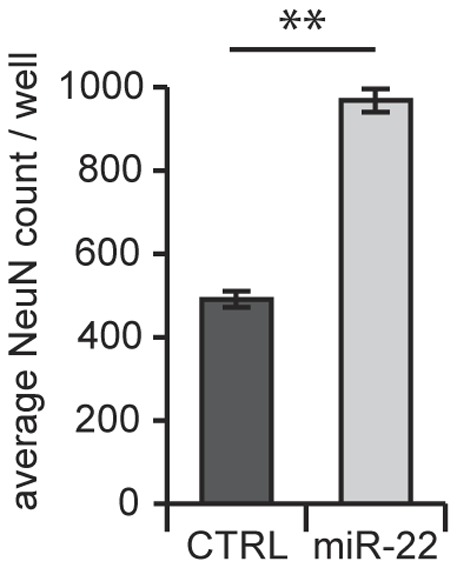
miR-22 is neuroprotective against long-term culture stress. Primary cortical neurons were infected with mir-22-expressing lentivirus on DIV10 and neuronal survival was compared to cultures not overexpressing miR-22 (CTRL) after 5 weeks. Graphs represent mean ± SEM, n = 15. ** represents p-value <0.01, by unpaired t-test.

We then asked whether miR-22 could prevent neurodegeneration in a non-HD-like condition. We thus assessed the effects of miR-22 against neurodegeneration due to long-term culture stress. This model relies on long-term culture of cortical neurons for >5 weeks, after which time a significant decrease in the number of neuronal cells begins to be observed [22 and D. Taylor, R. Moser, and R. Luthi-Carter, unpublished observations]. As in the experiment shown in Figure 4, we observed that initially the exposure to miR-22-expressing lentiviral vector decreased neuronal number (reflecting cell loss due to nonspecific toxicity of the lentiviral vector application). However, as hypothesized, expression of miR-22 increased neuronal long-term neuronal survival, as measured by NeuN-positive cell counts at 5 weeks *in vitro* (**Figure 5**).

### miR-22 also Targets Other Cell Death Regulators

**Figure 6:**
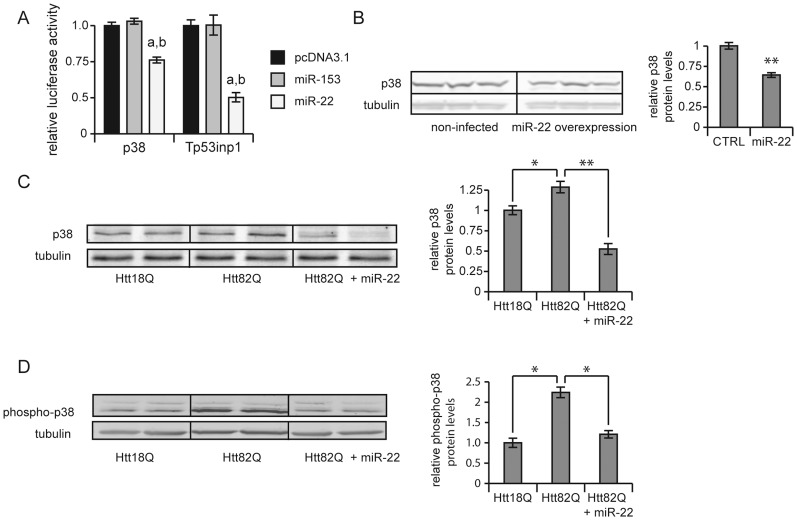
miR-22 downregulates protein levels of MAPK14/p38 and tp53inp1. A) Luciferase constructs expressing the *Renilla* luciferase gene under the control of the MAPK14/p38 or Tp53inp1 3′ UTRs were co-transfected in HEK293T cells with either an empty plasmid (pcDNA3.1), plasmid expressing a control miR-153 not predicted to target the 3′ UTR or a plasmid expressing miR-22. The intensity of *Renilla* luciferase luminescence was normalized to firefly luciferase luminescence, which was not under the control of a 3′ UTR. Graphs represent average values ± SEM, n = 6. a indicates p-value <0.01, by unpaired t test compared to the control sample transfected with the empty vector pcDNA3.1. b indicates p<0.01, by unpaired t test compared to the control sample transfected with a control microRNA, miR-146a or -153, not predicted to bind the 3′ UTR. B) Primary cortical neurons were infected with lentivirus expressing miR-22 and proteins were harvested 3 weeks post-infection. MAPK14/p38 protein levels in miR-22 overexpressing cells were compared to MAPK14/p38 protein levels in the non-infected control cells (CTRL). Protein levels were normalized to β-3 tubulin, which is not a predicted target of miR-22 and whose expression did not change with miR-22 treatment. Graphs represent quantification of the Western blot, error bars represent SEM, ** represents p-value <0.01, by unpaired t-test. CTRL – non-infected cells. C) and D) Primary striatal neurons were infected on DIV1 with lentiviral vectors encoding WT (Htt18Q) or mutant (Htt82Q) Htt171 fragments under the control of the TRE promoter and co-infected on DIV4 with vector encoding miR-22 and 2.5 weeks post-infection proteins were harvested. Protein levels were normalized to β-3 tubulin, which is not a predicted target of miR-22. Graphs represent quantification of the signals from the Western blot, error bars represent SEM. * represents p-value <0.05, ** represents p-value <0.01, by unpaired t-test.

Given that none of the HD-related targets explored above have been implicated in neuronal aging, we considered what other targets of miR-22 might be relevant for its broader neuroprotective effects. In so doing, we noted that miR-22 is also predicted by TargetScan to target the 3′ UTRs of the Tp53inp1 and MAPK14/p38 mRNAs, whose encoded proteins have known pro-apoptotic activities [23], [24]. We subsequently confirmed the targeting of these mRNAs by miR-22 using 3′ UTR luciferase assays, and also showed by Western blot that miR-22 overexpression in primary neurons decreases the cellular accumulation of MAPK14/p38 protein (**Figure 6A, B**). (We were unable to reliably detect Tp53inp1 protein in our rat primary cultures by Western blot, preventing us from assessing a possible effect of miR-22 on its accumulation.) We also assessed the effects of miR-22 on levels of MAPK14/p38 protein and its activation during neurodegeneration. These analyses showed that both the increased cellular levels of total MAPK14/p38 (**Figure 6C**) and activated phosphorylated MAPK14/p38 associated with neurotoxicity were abrogated by the overexpression of miR-22 (**Figure 6D**). These data support the conclusion that the inhibition of MAPK14/p38 activity comprises one of the neuroprotective capabilities of miR-22.

### miR-22 Inhibits Neuronal Apoptosis

**Figure 7:**
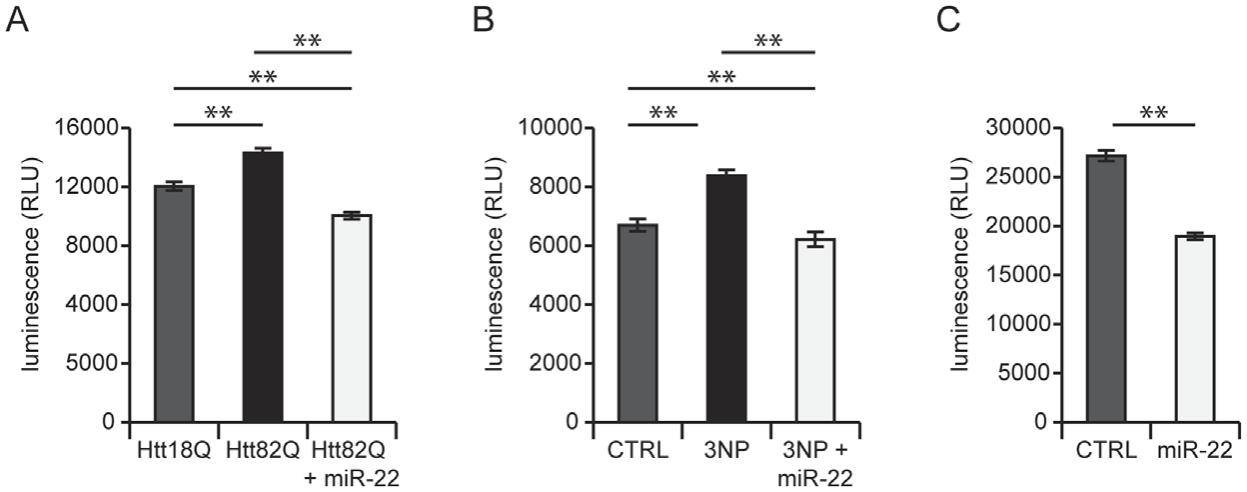
miR-22 decreases caspase activation preceding neuronal cell death. miR-22 was assessed for its effects on caspase 3/7 activities in primary striatal neurons in the following neurodegenerative conditions: A) expressing a fragment of mutant Htt (Htt82Q), B) treated with 150 µM 3-NP or C) long-term culture (to model aging). The miR-22-overexpressing condition is compared to the control with no miRNA overexpression (see text for rationale). Graphs represent mean ± SEM, ** represents p-value <0.01, by unpaired t-test, CTRL – non-infected cells.

Having determined that miR-22 inhibits the expression of pro-apoptotic proteins, we next sought to evaluate whether miR-22 inhibited downstream manifestations of apoptosis. We thus tested this hypothesis by measuring the effect of miR-22 overexpression on the activity of effector caspases (3 and 7). As expected, we observed significant activation of effector caspases in all models of neurodegeneration (Htt171-82Q, 3-NP, and aging), which in each case was inhibited overexpression of miR-22 (**Figure 7A, B, C**).

Taken together, the above results show a therapeutic potential of miR-22 mediated by both general anti-apoptotic effects (via targeting MAPK14 and Trp53inp1), and specific HD-related effects (via Rcor1, HDAC4, Rgs2 and Htt).

## Discussion

The dysregulation of miRNA expression has been implicated in the pathogenesis of a number of neurodegenerative diseases. In addition to being an integral part neuropathogenesis, miRNAs can serve as disease modulators. For example, upregulation of miR-206 has been shown to slow the progression of neurodegeneration in a mouse model of amyotrophic lateral sclerosis by promoting regeneration of neuromuscular synapses [9]. Since a single miRNA can have a broad effect by targeting tens or hundreds of genes, miRNAs may represent a new approach to polytherapy for complex diseases. Here, we have shown that increasing cellular levels of miR-22 might be one such approach for combating neurodegeneration.

We demonstrated that overexpression of miR-22 is neuroprotective in *in vitro* models of HD, which may be explained by miR-22′s targeting multiple genes previously implicated in HD pathogenesis. These include Rcor1 and HDAC4, which have been implicated in HD-related abnormalities in gene expression [11], [25]. Rcor1 is a transcriptional co-repressor in the REST/NRSF pathway, which regulates the expression of neuronal genes including brain-derived neurotrophic factor, an important neuronal plasticity modulator and pro-survival factor for striatal and cortical neurons. Histone hypoacetylation has also been observed in HD and histone deacetylase inhibitors can ameliorate disease phenotype in animal models of HD [26]–[28]. HDAC4 is of particular interest, given that it may be an important mediator of the effects of the HDAC inhibitor SAHA [14]. Not least of all, downregulation of Rgs2 expression is a consistent feature of human HD and multiple HD models, and our own recent findings have demonstrated that inhibition of Rgs2 is protective in HD though enhancement of ERK activity [12]. Moreover, direct or indirect inhibition of mutant Htt accumulation (albeit through a presently unknown mechanism) would be of clear benefit in HD. In addition to HD-specific pathways, miR-22 also demonstrated the ability of to inhibit apoptosis, as shown by its ability to decrease effector caspase activation. We propose that this effect may be mediated, at least in part, by its targeting pro-apoptotic genes MAPK14/p38 and Trp53inp1.

The targeting of general anti-apoptotic and HD-specific pathways, together with evidence that it is downregulated in HD brain, make miR-22 a particularly interesting strategy for combating HD. In addition, based on its anti-apoptotic properties and abilities to prevent neurodegeneration in non-Htt-based paradigms, it may also be interesting to consider its utility in other neurodegenerative conditions. Our encouraging *in vitro* results provide an excellent rationale for investigating whether miR-22 can also achieve neuroprotection *in vivo*. However, it is somewhat unclear whether direct manipulation of miR-22 overexpression would be the preferred strategy to achieve increased miR-22 levels. Such manipulation might be plausible if localized delivery to a small set of affected neurons is sufficient. Alternatively, exploring other ways by which miR-22 levels could be regulated by small-molecule drugs might be another therapeutic option.

### Conclusion

Our study demonstrates that enhancing miR-22 expression in neurons has a pronounced neuroprotective against a variety of medically relevant neuronal insults. These results support the perspective that enhancing miR-22 expression might comprise a rational therapeutic strategy for the treatment of HD and other neurodegenerative conditions.

## Methods

### Plasmids and Lentiviral Vectors

Lentiviral vectors encoding the first 171 amino acids of human Huntingtin containing 18 or 82 polyglutamine repeats under control of a tetracycline response element (TRE) regulated promoter (SIN-TRE-htt117-18Q/82Q-WPRE), the tetracycline transactivator (tTA) under the control of a phosphoglycerate kinase (PGK) promoter (SIN-PGK-tTA-WPRE), and the rat miR-22 gene (rno-miR-22) under the control of PGK promoter (SIN-PGK-miR-22-WPRE) were produced in human embryonic kidney 293T (HEK293T) cells with a four-plasmid system as described previously [29]. Rno-miR-22 gene was cloned together with the surrounding genomic region of total length 295 bp using the following primers: F-miR-22 cacccagagctaggttcagatggccagtgggtc and R-miR-22 ctgtaaagccgtcaccccagctgtccagac. SIN-PGK-miR-22-WPRE plasmid was also used to overexpress the miRNA by transfection in human embryonic kidney (HEK) T293 cells in 3′ UTR-luciferase assays.

### Primary Cultures and Lentiviral Infections

Primary cortical and striatal neuron cultures used for neuroprotection assays were prepared from striata (ganglionic eminences) or cerebral cortices of E16 rat embryos as described in [29]. This procedure has been demonstrated to yield a high majority of neuronal cells with some residual glial cells [17]. Cells were plated in 96-well plates, not using the wells in the most outer rows (rows A and H) and columns (1 and 8).

*In vitro* lentiviral models of HD were prepared as described in [17]. Primary striatal or cortical neuronal cultures were infected at one day *in vitro* (DIV) with lentiviruses encoding the first 171 amino acids of wild-type or mutant human Htt (SIN-W-TRE-Htt171-18/82Q, respectively). Htt-expressing lentiviruses were applied at a concentration of 25 ng p24 antigen/ml culture medium together with a vector encoding the tetracycline-controlled transactivator tTA1 under the control of PGK promoter (at a concentration of 40 ng p24/ml). At DIV4 neurons were infected with lentiviral expression constructs for rno-miR-22 (at a p24 concentration of 25 ng/ml). Three weeks after the infection of Htt constructs, cells were fixed and their survival assessed by NeuN counting. The transduction efficiency of primary neurons under these conditions has been shown to be ≥ 95% [17], [29].

### 3-Nitroproppionic Acid Toxicity Studies

Primary cultures of striatal neurons were prepared as in [29] and infected at DIV10 with lentiviral construct expressing rno-miR-22 (at a p24 concentration of 25 ng/ml). At DIV24 cultures were treated with 150 or 300 µM 3-nitropropionic acid (3-NP). Forty-eight hours later cells were fixed and their survival assessed by NeuN-positive cell counting.

### Cellular Model of General Neurodegeneration

Primary cultures of cortical neurons were prepared as in [16] and infected at DIV10 with a lentiviral vector expressing rno-miR-22 (at a p24 concentration of 25 ng/ml). Four weeks after lentiviral infection cells were fixed and their survival assessed by NeuN-positive cell counting.

### Immunolabeling and Microscopic Image Analyses

Cell cultures were washed with cold phosphate-buffered saline (PBS) and fixed with 4% paraformaldehyde (Fluka) for 15 min at RT. Cultures were subsequently washed with PBS and incubated in a blocking solution of PBS with 10% normal goat serum (Dakocytomation) and 0.1% Triton X-100 (Sigma). Cells were then incubated overnight at 4°C in blocking solution containing primary antibodies: mouse monoclonal anti-Neuronal Nuclei (NeuN) (1∶1000, Chemicon Millipore MAB377 clone A60), mouse monoclonal 2B4 anti-huntingtin (1∶500, Chemicon Millipore MAB5492). Secondary antibodies used goat anti-mouse IgG Alexa Fluor 488 or goat anti-mouse IgG Alexa Fluor 594 (1∶800, both from Invitrogen) were applied for 1 h at room temperature.

Images for neuroprotection assays were acquired with a BD Pathway 855 instrument and cell bodies or Htt-positive foci were counted with ImageJ software [30]. Images for quantitative analyses were acquired under non-saturating exposure conditions. Htt accumulation was quantified as numbers of Htt-positive foci of a mean fluorescence intensity >10 times the overall mean pixel intensity and ≥2 µm in diameter per well. These foci were observed in N171-82Q-expressing, but not N171-18Q-expressing cells [29].

### Immunoblotting

For analysis of protein expression cells were harvested in RIPA buffer (Sigma), containing protease inhibitor cocktail (Sigma). Proteins were separated on 10% SDS-polyacrylamide gels and transferred to nitrocellulose membranes. Membranes were blocked for one hour in 30% Odyssey® Blocking Buffer (LI-COR Biosciences), 0.1% Tween-20 in PBS followed by incubation in primary antibodies diluted in Blocking Buffer with 0.1% Tween-20 overnight at 4°C. Membranes were rinsed with 0.1% Tween-20/PBS followed by incubation with secondary antibody diluted in Blocking Solution for one hour at RT. After final rinses, blots were scanned with an Odyssey® Infrared Imager and densitometric measurements were obtained using Odyssey® software (LI-COR Biosciences). Primary and secondary antibodies comprised: rabbit polyclonal anti-tubulin (Sigma, 1∶2000), mouse monoclonal anti-MAPK14/p38α (Cell Signaling clone L53F8, 1∶1000), mouse monoclonal anti-phospho-MAPK14/p38α (Cell Signaling clone 28B10, 1∶1000), donkey anti-mouse IRDye 800CW (LI-COR Biosciences, 1∶15,000), donkey anti-rabbit IRDye 680 (LI-COR Biosciences, 1∶15,000).

### Luciferase Reporter Assays

Full length 3′ UTRs were cloned into the multiple cloning site of the psiCHECK™-2 vector (Promega, Cat. number C8021). 3′ UTR sequences were retrieved from TargetScan and primers were: F-HDAC4-3′ UTR: ataagtcgacaagctgctgttctctcctttgt, R-HDAC4-3′ UTR: taaagcggccgcgagggcaattaacgcagtcttt, F-Rcor1-3′ UTR: ttaagtcgacgctgcttctgtcctcgacgta, R-Rcor1-3′ UTR: taaagcggccgccatattttattacatcaatggcttg, F-Rgs2-3′ UTR: ctcgagaagccacagatcaccacggagccccatgctacatga, R-Rgs2-3′ UTR: gcggccgcagtattcaaaaaccacttcagag, F-mapk14-3′ UTR: aaaactcgaggcaccttgcttctgttct, R-mapk14-3′ UTR: gtaaagcctatttttcagattcgcggccgcaaaa, F-tp53inp1-3′ UTR: ctcgagctatggttgccttgaaattatc, R-tp53inp1-3′ UTR: gcggccgctgtaactctgggcagtgcaa.

5 ng of the psiCHECK™-2-3′ UTR construct was cotransfected with 500 ng of a lentiviral vector encoding miR-22 into human embryonic kidney 293T cells (HEK293T), and the luciferase activity was measured two days later using the Dual-luciferase reporter assay (Promega) according to the manufacturer’s instructions. Luminescence was measured on a TECAN GenioPro plate reader in the linear range of the instrument.

### Caspase Activity Assays

Caspase 3/7 activity assays were performed using Caspase-Glo ® 3/7 Assay by Promega following the manufacturers instructions. Briefly, the medium of cells cultured in 96-well plates was reduced to 50 µl/well and 50 µl of Caspase-Glo reagent was added. Cells were incubated with the reagent for 30 minutes and luminescence was measured on a TECAN GenioPro plate reader in the linear range of the instrument. Each condition was represented with 7 biological replicates. Caspase activity was measured at 24 hours for neurons treated with 3-NP, 16 days for cells expressing Htt171-82Q, and 4 weeks in the aging model.
